# Cellular senescence predicts treatment outcome in metastasised colorectal cancer

**DOI:** 10.1038/sj.bjc.6605784

**Published:** 2010-07-13

**Authors:** A M Haugstetter, C Loddenkemper, D Lenze, J Gröne, C Standfuß, I Petersen, B Dörken, C A Schmitt

**Affiliations:** 1 Medical Department of Hematology, Oncology and Tumor Immunology, Molekulares Krebsforschungszentrum der Charité – MKFZ, Charité – Universitätsmedizin Berlin, Augustenburger Platz 1, 13353 Berlin, Germany; 2 Institute of Pathology, Technische Universität München, Munich, Germany; 3 Department of Pathology, Campus Benjamin Franklin, Charité – Universitätsmedizin Berlin, Berlin, Germany; 4 Department of General, Vascular and Thoracic Surgery, Campus Benjamin Franklin, Charité – Universitätsmedizin Berlin, Berlin, Germany; 5 Department of Bioinformatics, Free University, Berlin, Germany; 6 Department of Pathology, Friedrich Schiller University, Jena, Germany; 7 Max-Delbrück-Center for Molecular Medicine, Berlin, Germany

**Keywords:** cellular senescence, colorectal cancer, *Ras* mutations, treatment outcome

## Abstract

**Background:**

Cellular senescence is a terminal cell-cycle arrest that occurs in response to activated oncogenes and DNA-damaging chemotherapy. Whether cancer cell senescence at diagnosis might be predictive for treatment outcome is unknown.

**Methods:**

A senescence index (SI) was developed and used to retrospectively correlate the treatment outcome of 30 UICC stage IV colorectal cancer (CRC) patients with their SI at diagnosis.

**Results:**

5-Fluorouracil/leucovorin-treated CRC patients achieved a significantly longer progression-free survival when presenting with SI-positive tumours before therapy (median 12.0 *vs* 6.0 months; *P*=0.044).

**Conclusion:**

Cancer cell senescence predicts treatment outcome in metastasised CRC. Prospective analyses of larger patient cohorts are needed.

In the past, many cancer studies sought to link quantitative assessments of tumour growth properties, namely proliferation and apoptosis, to treatment outcome, but the majority of these analyses produced negative results for various reasons. Although relevant as a prognosticator, the value of using the mitotic index to predict treatment outcome is very limited (see Schmitt, 2003, 2007; Brown and Attardi, 2005; Yerushalmi *et al*, 2010 and references therein for review). A third cancer-related growth condition is oncogene-induced senescence (OIS), a terminal cell-cycle arrest initiated by activated Ras-type oncogenes that functions as a tumour-suppressive barrier in pre-malignant lesions *in vivo* (Braig *et al*, 2005; Michaloglou *et al*, 2005; Collado and Serrano, 2010; Reimann *et al*, 2010). Oncogene-induced senescence is mediated through an oncogene-evoked DNA damage response (DDR) (Bartkova *et al*, 2005, 2006; Gorgoulis *et al*, 2005), thereby explaining why DNA-damaging chemotherapy produces senescence as well (Chang *et al*, 1999; Schmitt *et al*, 2002). Therapy-induced senescence (TIS) contributes to treatment outcome in pre-clinical models (Schmitt *et al*, 2002), and is detectable in patient cancer biopsies after neoadjuvant chemotherapy (te Poele *et al*, 2002; Roberson *et al*, 2005), but its long-term effect on clinical courses remains undetermined. We hypothesised that senescent cells present sporadically in untreated cancers at diagnosis may indicate an increased susceptibility to TIS, and, thus, might be associated with a superior clinical outcome. Because the gold-standard senescence assay, detection of the senescence-associated *β*-galactosidase (SA-*β*-gal) activity (Dimri *et al*, 1995), cannot be applied to formalin-fixed and paraffin-embedded (FFPE) clinical routine samples, we developed a senescence index (SI) based on FFPE-suitable immunohistochemical surrogate markers. We report here the first-time use of this SI in untreated human tumour specimens at diagnosis as a predictor for outcome to cancer therapy.

## Materials and methods

### Patients and tumour material

Snap-frozen or FFPE samples of normal colon mucosa, colon adenoma and colon carcinoma were collected, and informed patient consent was obtained for the anonymous use of the material. FFPE tumour samples from previously untreated 30 patients with UICC stage IV colorectal cancer (CRC) all of whom received a 5-fluorouracil/leucovorin (5-FU/LV)-based first-line regimen were subjected to senescence evaluation, reflecting the available archive material in our pathology department between the years 1996 and 2002 that met these criteria. Tissue material from patients suspected or diagnosed with a hereditary cancer syndrome or inflammatory bowel disease, or whose chemotherapy was terminated for reasons other than disease progression was excluded from this study. Clinical data sets were compiled in a retrospective and anonymous manner, and included gender, age, tumour location, carcinoembryonic antigen (CEA) serum levels, TNM classification, tumour grading and progression-free survival (PFS), defined as the time from first diagnosis until diagnosis of progressive disease (i.e. local recurrence of the primary lesion, or growth of metastases or occurrence of new metastatic lesions).

### Retroviral infection

IMR90 human fibroblasts (CCl-186 from American Type Culture Collection, Manassas, VA, USA) were stably transduced with the pBabe-Puro-H-*Ras*V12 retrovirus (provided by S Lowe) or an empty vector as a control, and selected in puromycin as described (Serrano *et al*, 1997; Braig *et al*, 2005). Cell pellets were snap frozen in liquid nitrogen or processed as FFPE pellets comparable to patient tissue samples.

### Immunohistochemical analysis

Cryosections (12 *μ*m) were acetone fixed and exposed to an anti-Ki67 primary antibody (M7240, 1 : 250 dilution; Dako, Glostrup, Denmark) for 30 min. Antibody binding was visualised by a chromogenic substrate in a streptavidin/alkaline phosphatase-amplified secondary antibody according to the manufacturer's recommendations (K0689, K0698, K0625, X3021, all from Dako).

FFPE sections (7 *μ*m) were subjected to heat-induced antigen retrieval before incubation with primary antibodies for 30 min. Primary antibodies against Ki67 (as above), heterochromatin protein 1*γ* (HP1*γ*; MAB3450, 1 : 250 dilution; Chemicon/Millipore, Billerica, MA, USA), phospho-activated ERK1/2 (p-ERK, no. 4376, 1 : 50 dilution; Cell Signaling Technology, Danvers, MA, USA), plasminogen activator inhibitor-1 (PAI-1 (i.e. Ncl-PAI-1), 1 : 20 dilution; Novocastra, Newcastle upon Tyne, UK) or cleaved caspase-3/Asp175 (no. 9661, 1 : 200 dilution; Cell Signaling Technology) were used, followed by the appropriate secondary antibody along with a streptavidin/alkaline phosphatase conjugate or streptavidin/peroxidase kit and suitable chromogenic substrates such as 3,3′-diaminobenzidine or Fast Red (K0690, K3468, K5005, all from Dako).

The formula and the cut-off values of the SI were generated based on the expression (i.e. the percentage of positive cells by immunostaining) of the three markers p-ERK, HP1*γ* and PAI-1 in low-proliferating areas (arbitrarily defined as <12 Ki67-positive in an area of 100 cells) in a learning set of five adenoma samples. For each of the three markers, a factor was generated that reflects the reciprocal percentage of cells that stained positive in such areas averaged over these five adenomas. This factor was used as a coefficient to equalise the marker's relative individual contribution to the SI. Moreover, a linear correction value was added to set the discrimination threshold to 0. Values between −1 and +1 were considered ‘non-conclusive’. Senescence index values of individual cancer samples were obtained in low-proliferating areas as well (see Figure 2 for further information on SI-related technical procedures).

### Senescence-associated *β*-galactosidase activity

Senescence-associated *β*-galactosidase activity was detected at pH 6.0 in cryo-preserved cells or tissue sections as described (Dimri *et al*, 1995; Braig *et al*, 2005). Cases were considered senescent if their mean percentage of SA-*β*-gal-positive cells was higher than the mean percentage of Ki67-positive cells.

### *Ras* mutation analysis

Genomic DNA was extracted from macro-dissected tumour areas of FFPE tissue sections using the QIAamp DNA Mini kit (Qiagen, Hilden, Germany). PCR amplification of the first exon of the *k-ras* gene was performed according to van den Brandt and colleagues (Brink *et al*, 2003). The resulting 179 bp PCR product was sequenced using the BigDye Terminator v1.1 Cycle Sequencing kit on a 3130 Genetic Analyzer (Applied Biosystems, Foster City, CA, USA). Hotspot codon 12 or 13 mutations were detected by comparison with the germ-line sequence.

### Statistical analysis

Statistical analyses (SPSS software package, release 17.0; SPSS, Munich, Germany) of Kaplan–Meier survival plots were based on the log-rank (Mantel–Cox) test; additional statistical comparisons used the Wilcoxon–Mann–Whitney test (to probe equal distribution of age, frequency of Ki67-reactive and cleaved caspase-3-positive cells, and serum CEA levels in patient subgroups), the Fisher's exact test (with respect to gender, tumour localisation, sites of distant metastasis (one *vs* more than one) and *Ras* mutations) and the *χ*^2^-test (regarding the pT and the pN status). *P*-values <0.05 were considered statistically significant.

## Results

**Figure 1:**
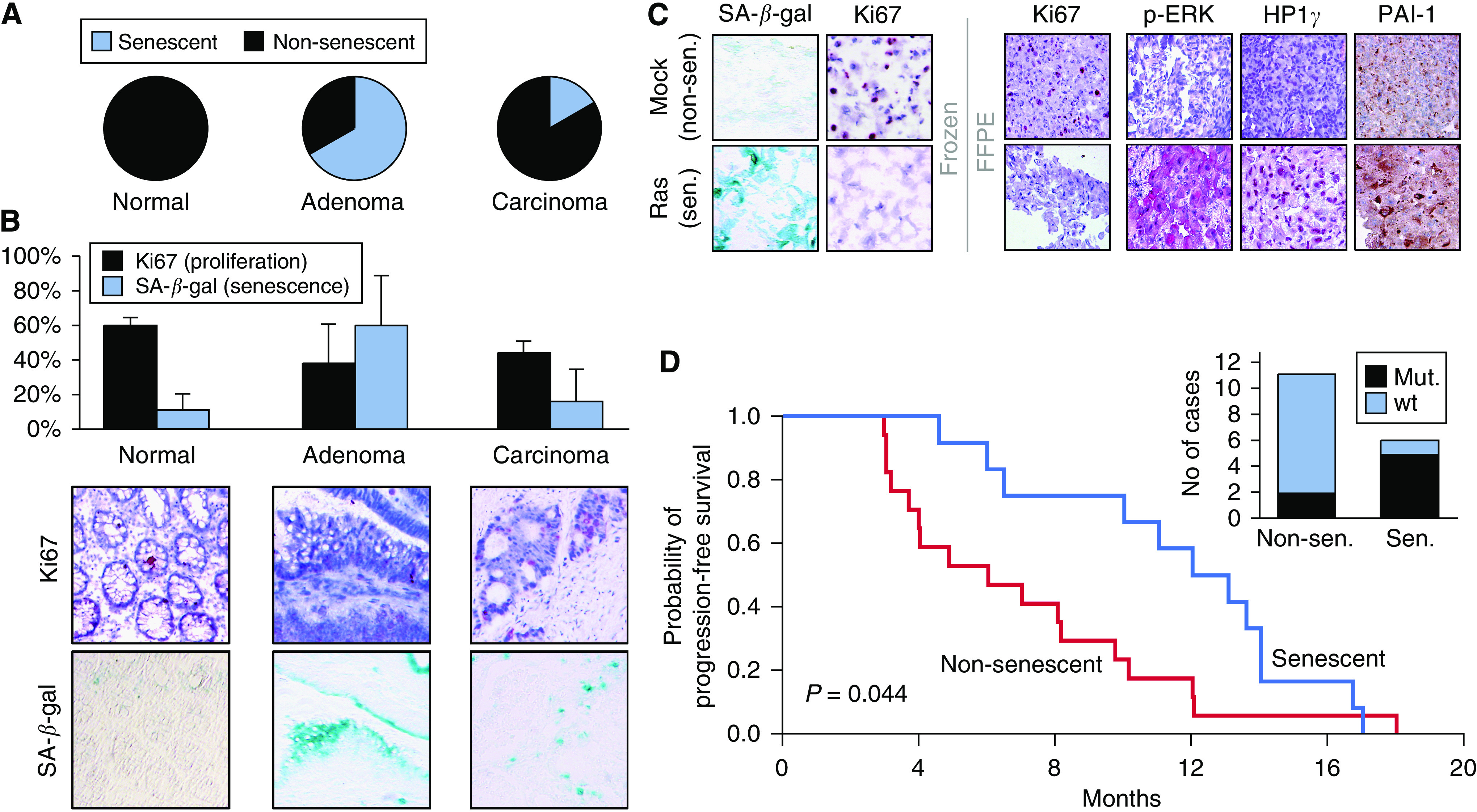
Stratification of stage IV CRC samples by a senescence index (SI) predicts PFS to first-line 5-FU/LV therapy. (**A**) Proportions of senescent cases in cryo-preserved colorectal tissue sections of the indicated groups (normal crypt mucosa (*n*=5, 0 senescent); adenoma (*n*=12, 8 senescent), invasive carcinoma (*n*=6, 1 senescent)). Note that 9 out of 12 adenoma and 2 out of 6 carcinoma cases would score ‘senescent’ if judged in low-proliferating areas only (see **D**). (**B**) Average frequency of senescent cells per group (as in **A**), measured as the percentage of SA-*β*-gal-positive (blue) cells, and compared to the rate of Ki67-positive (red nuclear staining) cells (top: error bars represent the standard deviation; bottom: matched areas of representative photomicrographs – a non-senescent normal crypt mucosa, a senescent adenoma and a formally non-senescent carcinoma (but showing numerous senescent cells)). Notably, in normal colorectal mucosa, only cells of the most differentiated luminal mucosa stain SA-*β*-gal-positive. (**C**) FFPE sections of pelleted Ras-infected senescent (sen.) human fibroblasts (SA-*β*-gal-positive and Ki67-negative frozen material as a reference) show much stronger immunoreactivity for p-ERK, HP1*γ* and cytoplasmic PAI-1 when compared with non-senescent (non-sen.) mock-infected fibroblasts. (**D**) SI values, based on the expression of these three markers, were obtained in 30 cases of stage IV CRC specimens at diagnosis, and used to stratify PFS following 5-FU/LV first-line chemotherapy (senescent (*n*=12; blue line) *vs* non-senescent (*n*=17; red line); one case scored ‘not conclusive’ (see Figure 2B)). Genomic sequencing in a subset of these 29 specimens identified K-*Ras* codon 12 or 13 mutations in 5 out of 6 senescent, but only 2 out of 11 non-senescent cases (inset). Note that a primary stratification by the K-*Ras* mutation status unveiled no significant differences in PFS (*P*=0.128).

**Figure 2:**
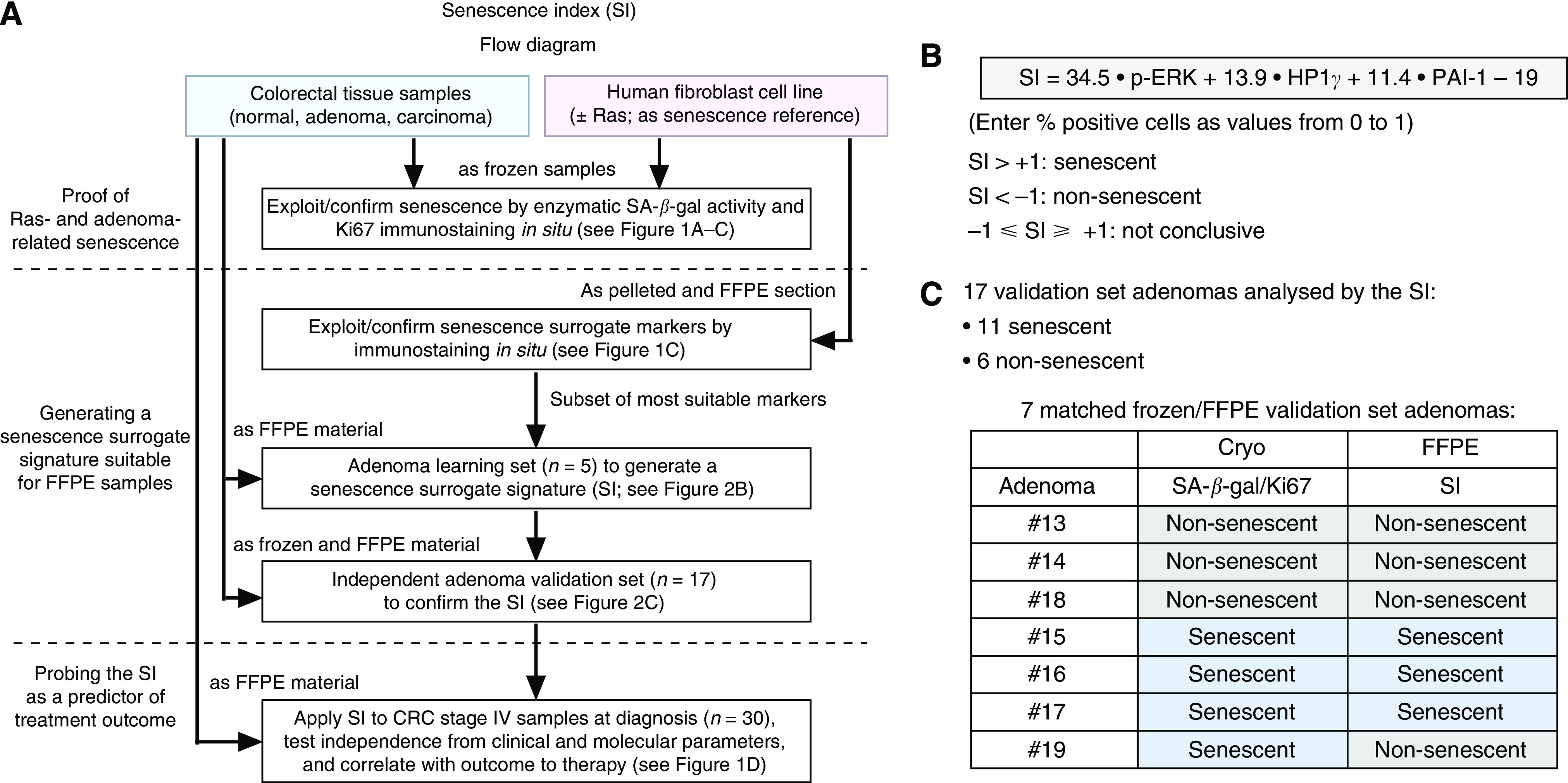
Generation and validation of the senescence index (SI). (**A**) Technical flow diagram of the generation and application of the SI in a *Ras*-transduced human fibroblast cell line and in colorectal tissue specimens. (**B**) Formula and cut-off values of the SI based on the expression p-ERK, HP1*γ* and PAI-1. Coefficients reflect the reciprocal average percentage of cells that stained positive for the respective marker in the learning adenomas to normalise the relative weight of the three markers. The correction value –19 was chosen to set the discrimination threshold at 0, with the range between −1 and +1 considered ‘non-conclusive’. (**C**) The majority of validation set adenomas is senescent when analysed by the SI. A set of seven matched samples (frozen and FFPE material from the same adenoma) underscores the high concordance between the SA-*β*-gal/Ki67- (see definition in Materials and Methods section) and the SI-based assignment.

We analysed 23 snap-frozen colorectal tissue samples of normal mucosa, adenomas and untreated invasive carcinomas by the SA-*β*-gal assay, complemented by immunostaining for the proliferation marker Ki67 and confirmed the reportedly high frequency of senescent cells in adenomas, leading to the classification of 8 out of 12 adenoma cases tested as senescent, which is well in line with their macroscopic presentation as polyps of often stable size for years (Figure 1A and B; see the flow diagram in Figure 2A for technical details) (Bartkova *et al*, 2006). Importantly, as previously reported for a subset of lymphoma, lung and breast cancer specimens at manifestation (te Poele *et al*, 2002; Roberson *et al*, 2005; Reimann *et al*, 2010), we detected a significant fraction of senescent cells within neoplastic epithelial areas of manifest colorectal carcinomas, possibly indicating a still available senescence programme at this full-blown cancer stage (Figure 1A and B).

Because the enzymatic SA-*β*-gal assay cannot be applied to FFPE routine samples, or substituted by a single marker, we tested protein expression levels of a panel of DDR mediators, cell-cycle regulators, chromatin-related proteins and others (Bartkova *et al*, 2006; Collado and Serrano, 2006) in a well-established OIS reference model system – sections of either snap-frozen or FFPE pellets of Ras-senescent *vs* proliferating fibroblasts – that allowed us to compare SA-*β*-gal staining and potential immunohistochemical surrogate markers in the same material side by side (Serrano *et al*, 1997). Using this fibroblast model system followed by first a learning and then a validation set of adenoma samples, we generated a senescence surrogate score (SI) based on the expression of p-ERK, HP1*γ* and PAI-1 – all of them previously linked to OIS (Serrano *et al*, 1997; Lin *et al*, 1998; Narita *et al*, 2003) – that recapitulated SA-*β*-gal reactivity and adenoma senescence (Figure 1C; Figure 2).

We obtained the SI in low-proliferating areas of 30 independent UICC stage IV CRC samples before any drug treatment, and assigned these cases, based on a cut-off value, to a (partly) senescent *vs* a non-senescent group. All patients in this retrospective analysis received a 5-FU/LV-based first-line regimen. Patients with senescent areas in their tumour biopsies experienced a significantly longer PFS with a median PFS of 12.0 *vs* 6.0 months when compared to the non-senescent group (Figure 1D). Notably, both groups did not show statistically significant differences regarding gender, age, pT and pN status as well as localisation of the primary tumour (colon *vs* rectum), extent of distant metastasis (one *vs* more than one site) or CEA serum levels at diagnosis, and showed indistinguishable rates of proliferation (i.e. averaged sample-wide Ki67 reactivity) and apoptosis (i.e. mean frequency of cleaved caspase-3-positive cells) (data not shown). Interestingly, K-*Ras* codon 12/13 mutations were found at a higher frequency (*P*=0.035) in the senescent group (Figure 1D, inset), suggesting that *Ras* mutations, if associated with a senescent phenotype, may not necessarily serve as a predictor of poor outcome, as reported by several studies in the past (Benhattar *et al*, 1993; Andreyev *et al*, 1998, 2001; Roth *et al*, 2010).

## Discussion

This is the first report linking cellular senescence at diagnosis to treatment outcome in cancer. Whether sporadic senescent cells in manifest tumours represent a few remainders of the pre-malignant condition, or indicate retained senescence susceptibility throughout the tumour is currently not clear. Our study data favour the latter explanation, postulating that TIS produced in response to DNA-damaging chemotherapy in senescence-capable tumour cells contributes to the overall outcome to therapy. Notably, an inverse correlation between the expression level of another senescence marker, macroH2A, and the risk of recurrence has recently been reported for lung cancer patients (Zhang *et al*, 2005; Sporn *et al*, 2009). Additional studies are certainly needed to clarify whether a greater extent of sporadic cancer cell senescence indeed translates into a higher frequency of TIS-positive cells *in situ* when analysed in re-biopsies a few days after chemotherapy. Moreover, a preserved pro-senescent cancer capability might be therapeutically exploitable by novel senescence-inducing strategies that do no longer damage DNA. The role of senescent cells at diagnosis as a novel predictor of treatment outcome should be further evaluated in larger prospective trials.
